# Transcriptome and Functional Analyses Revealed the Carboxylesterase Genes Involved in Pyrethroid Resistance in *Anopheles sinensis* (Diptera: Culicidae)

**DOI:** 10.3390/insects16090938

**Published:** 2025-09-05

**Authors:** Yiyun Wei, Xinyao Gu, Fengling Si, Xiaojie Chen, Liang Qiao, Hongxing Yan, Bin Chen

**Affiliations:** Chongqing Key Laboratory of Vector Control and Utilization, Institute of Entomology and Molecular Biology, College of Life Sciences, Chongqing Normal University, Chongqing 401331, China; weiyiyun1988@163.com (Y.W.); gxy576692235@163.com (X.G.); sifengling1217@163.com (F.S.); chenxiaojie092355@163.com (X.C.); qiaoliangswu@163.com (L.Q.); 18716278039@163.com (H.Y.)

**Keywords:** *Anopheles sinensis*, pyrethroid resistance, carboxylesterases, RNAi, functional analyses

## Abstract

Carboxylesterases (CCEs) have been demonstrated to be involved in pyrethroid resistance in insect species. This study aims to investigate CCE-mediated resistance mechanisms in *Anopheles sinensis*, a major malaria vector. Through comparative transcriptomics of a deltamethrin-resistant strain (CQ-LR) versus susceptible strain (WX-LS) of *An. sinensis*, we identified differentially expressed CCE genes across five developmental stages, five tissues, and three time points post-blood-meal. Four candidate genes (*AsAe9*, *AsAe10*, *AsAce2*, *AsUn5*) showed significantly upregulated expression. Subsequent qRT-PCR validation across four field-derived resistant strains (WX-LR, AH-LR, YH-LR, CQ-LR) and the susceptible strain confirmed significant upregulation of *AsAe9*, *AsAe10*, *AsAce1*, *AsAce2*, and *AsBe4* in more than two resistant populations. RNAi-based functional validation showed that silencing *AsAe10* or *AsBe4* in the WX-LR strain significantly decreased knockdown time and raised 24 h mortality upon diagnostic deltamethrin exposure, with *AsAe10* silencing having the strongest effect. This study identifies CCE genes involved in deltamethrin resistance in *An. sinensis*, providing valuable insights into the resistance mechanisms of pyrethroid and a theoretical basis for mosquito resistance management.

## Simple Summary

Pyrethroid resistance in *Anopheles sinensis*, a major malaria vector in Asia, severely compromises insecticide-based vector control. This study identified carboxylesterases as important factors in the mechanism of resistance to deltamethrin. Transcriptomic comparison of deltamethrin-resistant and susceptible strains revealed four upregulated carboxylesterase (CCE) genes. Based on prior evidence, we evaluated eight candidate CCEs, confirming significant upregulation of five genes (*AsAe9*, *AsAe10*, *AsAce1*, *AsAce2*, and *AsBe4*) in resistant strains via quantitative real-time PCR (qRT-PCR). The gene silencing mediated by RNA interference (RNAi) reduced the knockdown time and increased the mortality rates of resistant mosquitoes when exposed to deltamethrin. This study revealed carboxylesterase genes involved in pyrethroid resistance in *An. sinensis*, providing critical insights into resistance mechanisms and management strategies in mosquitoes.

## 1. Introduction

*Anopheles sinensis*, a primary vector for malaria and filariasis, exhibits extensive distribution across China and other East Asian nations [1,2,3]. The vivax malaria outbreak demonstrates strong epidemiological association with elevated vectorial capacity in *An. sinensis* for *Plasmodium vivax* [4]. Current strategies for controlling *An. sinensis* predominantly rely on pyrethroid-based interventions—insecticide-treated nets and indoor residual spraying—alongside supplemental organophosphate, organochlorine, and carbamate applications [5]. Alarmingly, the development of mosquito resistance to insecticides, particularly to pyrethroids, has compromised disease control efficacy [6,7,8,9]. Field-derived *An. sinensis* populations from Hainan, China, have developed resistance to DDT, cypermethrin, and malathion [10].

Target-site insensitivity and enhanced metabolic detoxification represent two principal mechanisms underlying insecticide resistance in mosquitoes [3,11]. Carboxylesterases, cytochrome P450s, and glutathione S-transferases in mosquitoes are typical enzymes correlated with pyrethroid resistance [11,12,13,14,15]. In recent years, our research has focused on *An. sinensis* to elucidate the mechanisms of insecticide resistance. Multiple gene families in mosquitoes have been confirmed to be involved in insecticide resistance mechanisms [16,17,18,19,20,21], including cytochromes P450, glutathione S-transferases, ATP-binding cassette transporters, and carboxylesterases. Carboxylesterases, members of the α/β-hydrolase fold superfamily [22], mediate specific hydrolysis of carboxyl ester bonds in organophosphates and pyrethroids [23,24,25]. For example, carboxylesterase Ha006a facilitates metabolic detoxification of pyrethroids (fenvalerate, λ-cyhalothrin, and deltamethrin) and organophosphates (paraoxon ethyl, profenofos, and chlorpyrifos) in *Helicoverpa armigera* [26]. Similarly, carboxylesterase RpCarE can hydrolyze isoprocarb and cyhalothrin in *Rhopalosiphum padi* [27]. Notably, Boest1 contributes to malathion and bifenthrin detoxification in *Bradysia odoriphaga* [28].

Gene mutation and overexpression represent two primary mechanisms for carboxylesterase-mediated pyrethroid resistance [29]. Specific mutations significantly enhance hydrolysis efficiency: *CarE001C* (H423I/R322L) in *H. armigera* elevates fenvalerate hydrolysis, while *E3* (W251L/F309L) in *Lucilia cuprina* increases permethrin degradation [30,31]. In pyrethroid-resistant *Musca domestica*, *MdαE7* mutations are accompanied by elevated expression and gene amplification, enhancing pyrethroid hydrolysis [32,33]. Overexpression of carboxylesterase genes further contributes to pyrethroid resistance. Upregulated expression of esterase genes in fenvalerate-resistant *H. armigera* midgut/fat body correlates with enhanced activity [34]. Constitutive/inductive *PxαE14* overexpression facilitates multi-pyrethroid detoxification in *Plutella xylostella* [35]. Studies have revealed significantly elevated carboxylesterase expression in pyrethroid-resistant mosquito populations, exemplified by upregulated *CPIJ018231*, *CPIJ018232*, and *CPIJ018233* in permethrin-resistant *Culex quinquefasciatus* [36], overexpressed *CCEae3a* in deltamethrin-resistant *Aedes aegypti* [37], and increased *COEAE1F* expression in deltamethrin-resistant *Anopheles gambiae* [38]. Moreover, increased esterase activity was observed in deltamethrin-resistant *Cx*. *pipiens pallens* and *Aedes aegypti* strains [39,40]. Correlative analysis by Chang et al. identified carboxylesterases as contributors to deltamethrin/permethrin resistance in *An. sinensis* [3], while Wu et al. found upregulated carboxylesterase genes in deltamethrin-resistant strains [16]. Collectively, these findings establish a correlation between pyrethroid resistance and carboxylesterase overexpression in mosquitoes, suggesting potential involvement of *An. sinensis* carboxylesterases in pyrethroid resistance. However, identification and functional analyses of specific pyrethroid resistance-associated carboxylesterase genes in *An. sinensis* remain limited.

In this study, comparative transcriptomics of susceptible and resistant *An. sinensis* strains were utilized to identify CCE genes exhibiting upregulated expression. Based on these findings and prior evidence of CCE-mediated resistance mechanisms, we identified candidate CCE genes of *An. sinensis* associated with deltamethrin resistance. Functionally, qRT-PCR was performed to confirm five upregulated genes across different resistant strains, and their causal roles in resistance were further elucidated through RNA interference (RNAi) and bioassays. These results enhance our mechanistic understanding of CCE-facilitated pyrethroid resistance and provide a theoretical basis for optimizing mosquito control strategies.

## 2. Materials and Methods

### 2.1. Mosquito Strains

The susceptible strain WX-LS, originally sourced from Wuxi, Jiangsu Province, China, has been maintained at the Institute of Entomology and Molecular Biology, Chongqing Normal University, China, under insecticide-free conditions. The resistant strain WX-LR was derived from WX-LS through deltamethrin selection. The pyrethroid-resistant strains CQ-LR, AH-LR, and YN-LR were established as near-isogenic lines via backcrossing for over ten generations between laboratory-susceptible strains and resistant populations collected from Chongqing, Anhui, and Yunnan, China, respectively. All five *An. sinensis* strains were reared separately under controlled conditions of 27 ± 1 °C and 70% ± 10% relative humidity. The susceptibility of adult female mosquitoes was evaluated three days post-emergence using the WHO standard bioassay protocol with filter papers impregnated with a diagnostic concentration of 0.05% deltamethrin (CAS: 52918-63-5, Sigma-Aldrich, St. Louis, MO, USA) [5]. The LC_50_ of deltamethrin for the laboratory-susceptible strain was 0.0067 mg/L. After a one-hour exposure to 0.05% deltamethrin-treated filter papers, the laboratory-resistant strains exhibited mortality rates of 14.29% for WX-LR, 13.33% for YN-LR, 17.14% for CQ-LR, and 11.43% for AH-LR following a 24 h recovery period.

### 2.2. Identification of Pyrethroid Resistance-Associated CCEs Using RNA-Seq

Transcriptomic analysis was performed on *An. sinensis*, encompassing both susceptible and resistant strains. Specimens were procured from diverse developmental phases, including fourth-instar larvae, female and male pupae, and adult females and males. Additionally, tissues from adult mosquitoes, namely the antennae, cuticle, fat body, Malpighian tubules, and midgut, were also collected. For blood-fed females, samples were gathered at 1, 3, and 12 h post-feeding. Three biological replicates were used to ensure reliability. The sequencing was conducted by Novogene Co., Ltd. (Beijing, China) using the Illumina HiSeq™ 2000 platform (Illumina, San Diego, CA, USA). Subsequently, following previously described methods [41,42], the data were analyzed by the Institute of Entomology and Molecular Biology, Chongqing Normal University. CCE genes associated with pyrethroid resistance in *An. sinensis* were identified based on the transcriptional profiles of distinct developmental stages, tissues, and blood-fed females from both deltamethrin-resistant (CQ-LR) and susceptible (WX-LS) strains. The sequencing reads were aligned to the *An. sinensis* genome using TopHat [43]. The expression levels of the CCE genes were quantified in FPKM (fragments per kilobase of transcript per million mapped reads) by employing Cufflinks [44]. Additionally, Cufflinks was utilized to assess the differential expression of transcripts between the resistant and susceptible strains. A gene was deemed significantly differentially expressed if it satisfied the following criteria: | log_2_ (fold change in FPKM) | ≥ 1 and *p*-value ≤ 0.05. These thresholds enabled the identification of potential CCE genes implicated in pyrethroid resistance. The differential expression patterns of the CCE genes were visualized using GraphPad Prism 10.0 software (GraphPad Software Inc., San Diego, CA, USA).

### 2.3. qRT-PCR Verification of Pyrethroid Resistance-Associated CCE Genes

Previous research has demonstrated that five genes (*CarE001G*, *MdαE7*, *Pxαe28*, and *Ldace1*) in *H. armigera*, *M. domestica*, *P. xylostella*, and *Leptinotarsa decemlineata* are involved in insecticide metabolism or are resistance-conferring [45,46,47,48]. Their corresponding homologs in *An. sinensis* have been identified as *AsBe4*, *AsAe4*, *AsUn4*, and *AsAce1.* These genes, along with the genes identified through RNA-seq analysis in this study, were selected as candidate target genes for validation via qRT-PCR. Total RNA was extracted using the Trizol Reagent Kit (Thermo Fisher Scientific Inc., Waltham, MA, USA) in accordance with the manufacturer’s instructions. The cDNA template was synthesized from the total RNA using the PrimeScript™ RT Reagent Kit with gDNA Eraser (TaKaRa, Biotech Co., Ltd., Dalian, China). The qRT-PCR assays were conducted using an iTaq™ SYBR^®^ Green Supermix (Bio-Rad, Hercules, CA, USA). Each treatment was replicated three times biologically. The *ribosomal protein L49* (*RPL49*) of *An. sinensis* was employed as the reference gene. The primers for qRT-PCR were designed using Primer Premier 5.0 (Supplementary Files S1). The relative expression levels were determined using the 2^−ΔΔCt^ method [49].

### 2.4. RNAI-Based Validation of Pyrethroid Resistance-Related CCE Genes

To elucidate the involvement of specific CCE genes in the pyrethroid resistance of *An. sinensis*, RNA interference (RNAi) was employed in conjunction with deltamethrin bioassays on female mosquitoes from the WX-LR strain. Primers for dsRNA synthesis were designed based on the cDNA sequences of the five CCE genes and *enhanced green fluorescent protein* (EGFP) using Primer Premier 5.0 (Supplementary Files S1). The dsRNA fragments were synthesized in vitro using the T7 RiboMAX Express RNAi System (Promega (Beijing) Biotech Co., Ltd., Beijing, China), with *EGFP* dsRNA serving as a negative control. The dsRNA concentrations were measured using Nano-Drop™ 1000 (Thermo Fisher Scientific Inc., Waltham, MA, USA). An 800 ng amount of the dsRNA fragments was injected into the hemocoel at the abdomen region between the second and third segment of the pupa at 10 h post-pupation of *An. sinensis*. The efficiency of gene silencing by dsRNA was evaluated using qRT-PCR in female adult mosquitoes three hours after emergence. The bioassay procedure adhered to the WHO standard insecticide susceptibility testing protocol [5]. Vigorous female adults were transferred to holding tubes for 1 h pre-exposure. Moribund/dead mosquitoes were discarded. Paper impregnated with 0.05% deltamethrin was secured to the inner surface of the exposure tubes. Following retraction of the sliding mesh partition, mosquitoes were gently blown into exposure tubes. Mosquitoes were exposed for 1 h with knockdown counts recorded at 10 min intervals. Survivors were transferred to holding tubes with 10% sugar water for 24 h recovery prior to mortality assessment. A mosquito was classified as dead or knocked down if it was immobile or unable to stand or take off. The knockdown curves of each treatment were fitted and the half-knockdown time (KT_50_) was calculated using GraphPad Prism 10.0 software.

### 2.5. Statistical Analysis

In this study, the disparities in gene expression levels and biological assay data across diverse treatments were analyzed. One-way analysis of variance (ANOVA) was initially conducted to assess the overall differences in expression levels among the distinct populations or developmental stages of *An. sinensis*, and Tukey’s multiple comparison tests were employed to discern the pairwise differences. The difference in knockdown rates between the control and treatment groups during the 1 h exposure to deltamethrin was analyzed using the Log-rank (Mantel–Cox) test. These analyses were performed using GraphPad Prism version 10.0 software.

## 3. Results and Discussion

### 3.1. Identification of Carboxylesterase Genes Associated with Pyrethroid Resistance in An. sinensis

#### 3.1.1. Identification of CCEs via Comparative Transcriptomics

**Figure 1:**
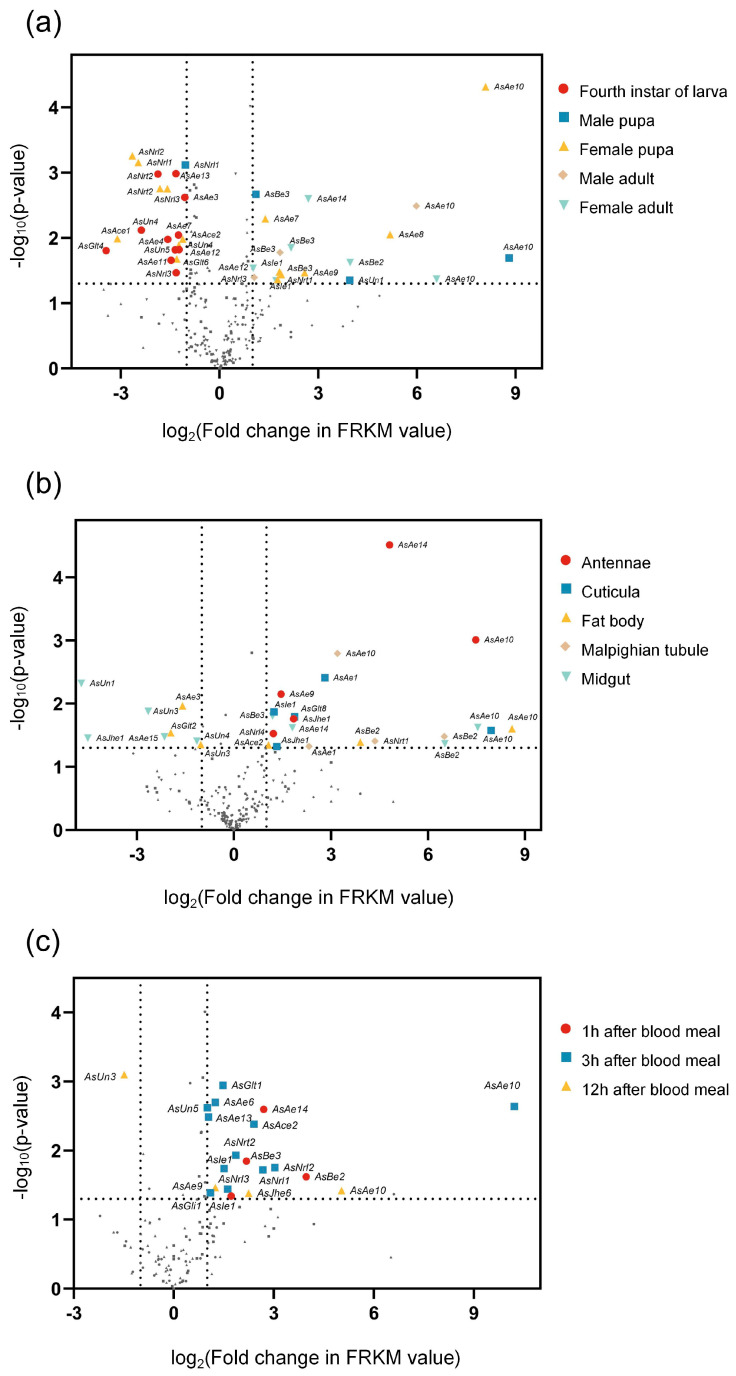
RNA sequencing analyses of differentially expressed *CCE* genes in deltamethrin-resistant *An. sinensis* strain (CQ-LR) versus susceptible strain (WX-LS). (**a**) Developmental stage-specific expression; (**b**) tissue-specific expression; (**c**) temporal dynamics of gene expression post-blood meal. Vertical dotted lines mark the ±1 of log_2_ (fold changes in FPKM value) values on the *x*-axis, and horizontal dotted lines denote the *p*-value = 0.05 of −log_10_ (*p*-value) on the *y*-axis. Criteria for significantly upregulated genes: |log_2_ (fold change in FPKM)| ≥ 1 and *p*-value ≤ 0.05.

The overexpression of esterase genes in mosquitoes facilitates their insecticide resistance [12,36,37,50,51]. In this study, RNA-seq was employed to profile differential expression of *CCE* genes in *An. sinensis* across five developmental stages, five tissue types, and three post-blood meal time points in paired deltamethrin-resistant (CQ-LR) and susceptible (WX-LS) strains. Developmental stage-specific profiling revealed that *AsAe10* and *AsBe3* exhibited significantly upregulated expression in four stages, excluding the fourth instar of larva (Figure 1a). The majority of overexpressed genes belonged to the *Ae* or *Be* classes, including *AsAe9*. In contrast, the measured *CCE* genes in the fourth larva exhibited significant downregulation in expression levels (Figure 1a). Tissue-specific profiling identified significantly upregulated *CCE* genes in *An. sinensis*: five in both the antennae and cuticle, four in the Malpighian tubules and midgut, and three in the fat body (Figure 1b). *AsAe10* exhibited significant overexpression across all five examined tissues, whereas *AsBe2* showed significant upregulation in three tissues (Figure 1b). Widespread upregulation was also observed for *Ae*-class genes, with *AsAe9* showing specific overexpression in antennae. The newly identified *AsAce2* exhibited upregulated expression in the fat body (Figure 1b). Specific temporal patterns of *CCE* gene upregulation emerged post-blood meal: four genes at 1 h, twelve genes at 3 h, and three genes at 12 h (Figure 1c). *Asle1* and *AsAe10* exhibited significant upregulation across two distinct temporal intervals. *AsAce2* and *AsUn5* had significant overexpression at 3 h after blood meal (Figure 1c). As shown in Figure 1, *AsAe9* and *AsAe10* exhibited significant upregulation across the three experiments, and *AsAce2* was overexpressed in both tissue-specific and post-blood meal assays. Notably, the homolog of *AsAe10* in *H. armigera*, *CarE001H*, has been functionally validated as a key gene involved in pyrethroid detoxification [52]. Similarly, the ortholog of *AsAce2* in *P. xylostella* (*Pxαe18*) plays an analogous role in detoxification processes [46]. Therefore, we preliminarily identify *AsAe9, AsAe10*, *AsAce2*, and *AsUn5* as genes associated with deltamethrin resistance in *An. sinensis*.

Developmental stage- and tissue-specific upregulation of carboxylesterase genes is involved in resistance mechanisms in insects. For instance, in a pyrethroid-resistant strain of *Cx. quinquefasciatus*, two esterase genes exhibit upregulated expression levels in both larval and adult stages relative to the susceptible strain [15]. Conversely, another study revealed that resistant larvae—but not adults—of the same species demonstrate significant overexpression of three distinct carboxylesterase genes [36]. This study proposes that constitutive upregulation of *CCE* genes in pupal and adult stages of the resistant *An. sinensis* strain reflects stage-specific selection pressure. Given that insecticide treatments target advanced developmental stages, these alterations in expression profiles could be associated with adaptive genetic responses to insecticide exposure, thereby driving resistance evolution. The midgut and fat body are typically organs for tissue-specific carboxylesterase expression, which is critical for metabolic detoxification [53,54,55,56,57,58]. In *P. xylostella*, the fipronil resistance-associated carboxylesterase genes *Pxae22* and *Pxae31* exhibit upregulated expression in fourth-instar larvae and the midgut [56]. Wu et al. documented 1.9-fold elevated esterase activity in a fenvalerate-resistant *H. armigera* strain (1690-fold resistance)**,** concomitant with 2-to-90-fold upregulated expression of four esterase genes in the midgut and fat body tissues relative to a susceptible strain [34]. Our earlier investigation revealed spatiotemporal overexpression patterns of pyrethroid resistance-associated *P450* genes in *An. sinensis*: elevated in resistant strains specifically within pupae/adults, the adult midgut/cuticle, and at 1- and 3 h post-blood meal [57]. Malpighian tubule-specific gene expression in mosquitoes facilitates dual roles in blood meal digestion and xenobiotic detoxification [58]. Reports indicate that *Anopheles* with pyrethroid resistance mediated by *kdr* mutations or *P450* overexpression exhibit altered blood-feeding capabilities [59,60]. Conversely, following a single blood meal, pyrethroid-resistant *An. funestus* exhibits significantly enhanced resistance to permethrin [61]. Researchers consider that insecticide detoxification mechanisms involved in resistance may be activated by a prior blood meal, thereby enhancing the resistance phenotype before insecticide exposure. Moreover, the blood meal sustains DDT and permethrin resistance during mosquito development, likely due to blood meal-induced elevation in glutathione S-transferase activity [62]. We hypothesize that, compared to the susceptible strain, significant blood-induced upregulation of carboxylesterase expression in the resistant mosquito strain may enhance blood meal metabolism. This could boost their fitness and stabilize insecticide resistance inheritance. Consequently, we measured carboxylesterase expression post-blood feeding in both resistant and susceptible mosquitoes in this study. Our integrated transcriptomic approach, validated in *An. gambiae*, *An. sinensis*, and *M. domestica* [38,63,64], consistently identifies carboxylesterase genes as important actors in pyrethroid resistance. Notably, transcriptomic analysis of the deltamethrin-resistant *An. sinensis* strain established by Zhou et al. did not detect overexpressed *CCE* genes [64]. This discrepancy with our findings is likely attributable to the strain’s geographic origin (Hubei, China) and differential resistance gene dominance.

#### 3.1.2. Validation of Differential Expression for CCEs via qRT-PCR

Many studies on mosquito resistance mechanisms validate the necessity for qRT-PCR confirmation of expression differences in candidate genes between resistant and susceptible strains [15,16,57]. qRT-PCR has been previously employed to identify pyrethroid resistance-associated *P450* and *GST* genes in *An. sinensis* [19,57]. The differential expression of eight genes—comprising four previously reported genes (*AsAe4*, *AsAce1*, *AsBe4*, *AsUn4*) and four genes newly identified in this study (*AsAe9*, *AsAe10*, *AsAce2*, *AsUn5*)—was quantified via qRT-PCR in deltamethrin-resistant and susceptible *An. sinensis* strains.

**Figure 2:**
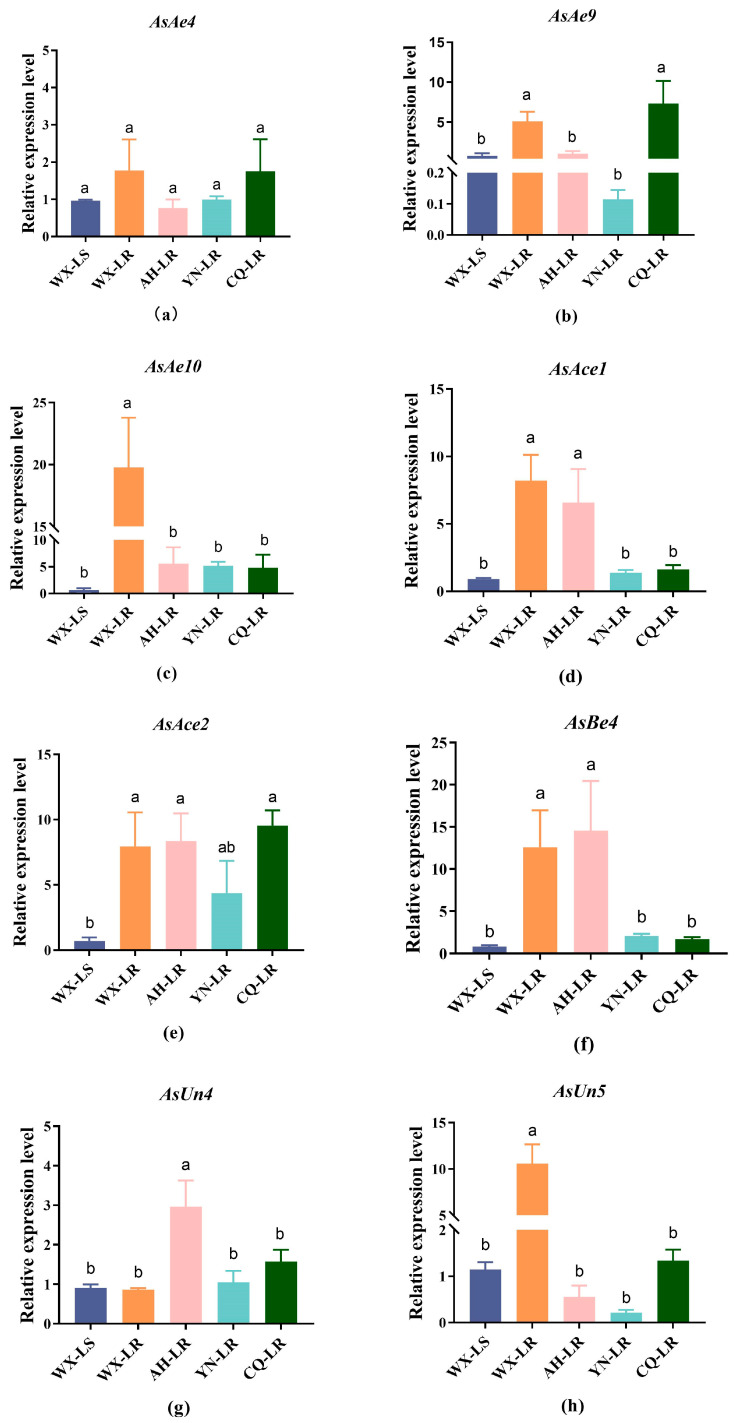
Validation of expression levels for eight selected carboxylesterase genes in deltamethrin-resistant (WX-LR, AH-LR, YN-LR, CQ-LR) and susceptible (WX-LS) *An. sinensis* strains. (**a**) *AsAe4*; (**b**) *AsAe9*; (**c**) *AsAe10*; (**d**) *AsAce1*; (**e**) *AsAce2*; (**f**) *AsBe4*; (**g**) *AsUn4*; (**h**) *AsUn5*. The genes’ relative expression levels are presented as the mean ± SD (standard deviation) from three biological replicates with three technical replicates each. Columns marked with the different lowercase letters indicate significantly differential expression across the strains (*p* < 0.05).

While *AsAe4* showed no significant upregulation in four *An. sinensis*-resistant strains compared with the susceptible WX-LS strain (Figure 2), its homolog *MdαE7* in resistant *M. domestica* is involved in permethrin detoxification [47,65]. Additionally, the *P. xylostella* homolog *PxEst-6* exhibits competence in binding and metabolizing bifenthrin, cyfluthrin, cypermethrin, and λ-cyhalothrin [66]. This functional difference likely reflects species-specific variations in pyrethroid detoxification metabolism mediated by the target gene. Significant upregulation of *AsAe9*, *AsAce1*, and *AsBe4* was detected in two *An. sinensis* resistant strains (Figure 2). *Ace1* shows upregulated expression in a pyrethroid-treated *L. decemlineata* strain, which may be implicated in insecticide resistance mechanisms [45]. A homolog of *AsBe4* (carboxylesterase *CarE001G* in *H. armigera*) exhibits metabolic activity toward β-cypermethrin, λ-cyhalothrin, and fenvalerate in vitro [48]. *AsBe4* (corresponding to *Bemisia tabaci* carboxylesterase *BTbe5*) demonstrates upregulation following imidacloprid exposure, and RNAi-mediated gene silence functionally validates its role in the insecticidal mechanism [67]. Elevated relative expression of *AsAe10* was observed across all four resistant strains, especially in the WX-LR strain (Figure 2), consistent with RNA-seq data from this study. The homolog of *AsAe10* in *H. armigera* (*CarE001H*) has been functionally validated as a key metabolic detoxification gene involved in pyrethroid catabolism [52]. *CCEae3A* (homolog of *Ae10*) exhibits significant upregulation in the *Ae. aegypti* temephos-resistant strain, and its overexpression in transgenic *An. gambiae* confers resistance to α-cypermethrin [51]. *AsAce2* exhibited significantly upregulated expression in four *An. sinensis* resistant strains (Figure 2). Its homolog *Pxae18* is overexpressed in chlorpyrifos-resistant *P. xylostella*, with RNAi confirming resistance involvement [46]. *AsUn4 and AsUn5* exhibited significant upregulation in the AH-LR and WX-LR strains, respectively (Figure 2). The homolog of *AsUn4* in *P. xylostella* (*PxαE28*) exhibits significant upregulation in chlorpyrifos-resistant strains, and RNAi-mediated gene silencing restores insecticide susceptibility, confirming its functional role in resistance [46]. Consequently, based on comparative transcriptomics and qRT-PCR validation, *AsAe9*, *AsAe10*, *AsAce1*, *AsAce2*, and *AsBe4* were selected as deltamethrin resistance-associated carboxylesterase genes for subsequent functional analysis.

### 3.2. Function Analysis of Carboxylesterase Genes in Pyrethroid Resistance of An. sinensis

#### 3.2.1. Determination of Time Points for CCE Silencing

**Figure 3:**
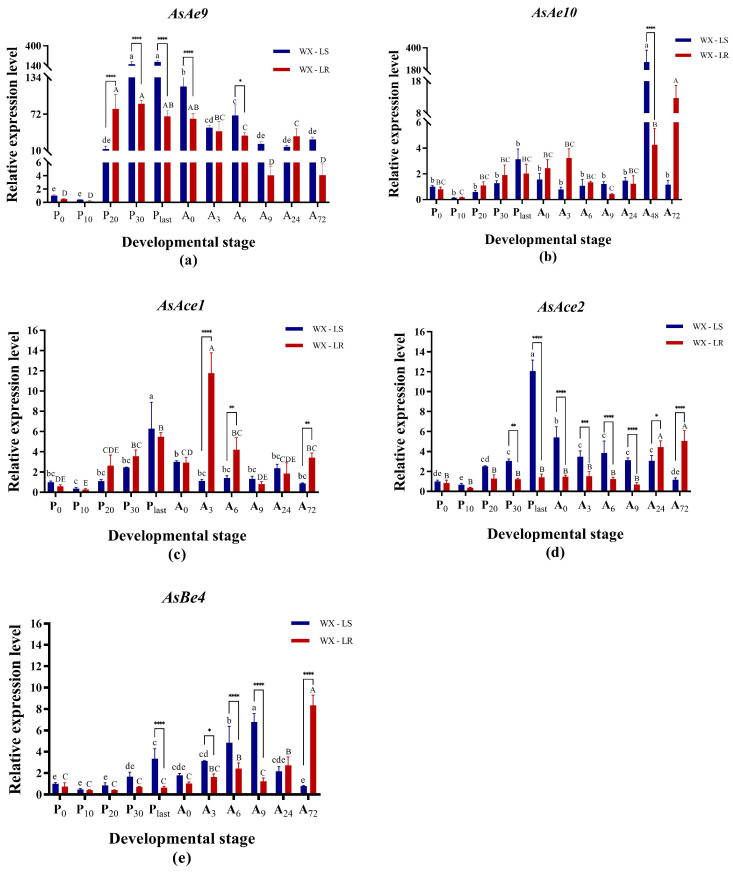
Developmental stage-specific expression profiling of five carboxylesterase genes in deltamethrin-resistant and susceptible *An*. *sinensis* strains. (**a**): *AsAe9*; (**b**) *AsAe10*; (**c**) *AsBe4*; (**d**) *AsAce1*; (**e**) *AsAce2*. The relative expression levels of genes were normalized to 0 h post-pupation of WX-LS, and are shown as the mean ± SD from three biological replicates in qRT-PCR analysis. Columns marked with homogeneous uppercase (for WX-LS) or lowercase (for WX-LR) letters indicate no significant difference in expression across developmental stages (*p* > 0.05). Asterisks indicate significant inter-strain differential expression at matched timepoints (*: *p* < 0.05; **: *p* < 0.01; ***: *p* < 0.001; ****: *p* < 0.0001). Pupal phases: P_0_ (0 h post-pupation), P_10_ (10 h post-pupation), P_20_ (20 h post-pupation), P_30_ (30 h post-pupation), P_last_ (terminal pupal stage). Adult stages: A_0_ (0 h post-emergence, newly emerged adults), A_3_ (3 h post-emergence), A_6_ (6 h post-emergence), A_9_ (9 h post-emergence), A_24_ (24 h post-emergence), A_72_ (72 h post-emergence).

Functional validation was performed through RNAi silencing to confirm the involvement of *AsAe9*, *AsAe10*, *AsAce1*, *AsAce2*, and *AsBe4* in pyrethroid resistance in *An. sinensis*. To determine the optimal developmental stage for dsRNA injection, the specific expression profiles of these five genes in pupae and adults in both pyrethroid-resistant (WX-LR) and susceptible (WX-LS) strains were acquired using qRT-PCR (Figure 3). All five genes exhibited expression through the pupal and adult stages, but had various expression levels across the different periods. In the WX-LS strain, *AsAe9*, *AsAce1*, and *AsAce2* exhibited maximal relative expression levels at P_last_ (terminal pupal stage), whereas *AsAe10* and *AsBe4* demonstrated divergent peak timing (Figure 3). In the WX-LR strain, three target genes (*AsAe10*, *AsAce2*, *AsBe4*) achieved peak expression at A_72_ (adults 72 h post-emergence). Compared to the susceptible strain, all five genes had significant upregulation in at least one adult stage of WX-LR (*p* < 0.05), with three genes concurrently overexpressed during pupal development (Figure 3). The five genes in both strains exhibited the lowest expression levels at the P_10_ stage (10 h post-pupation), with no significantly differential expression between strains (*p* > 0.05, Figure 3). This expression convergence established P_10_ as the optimal developmental stage for dsRNA microinjection in subsequent RNAi-mediated functional validation.

#### 3.2.2. Functional Analysis of CCE Genes in Pyrethroid Resistance

**Figure 4:**
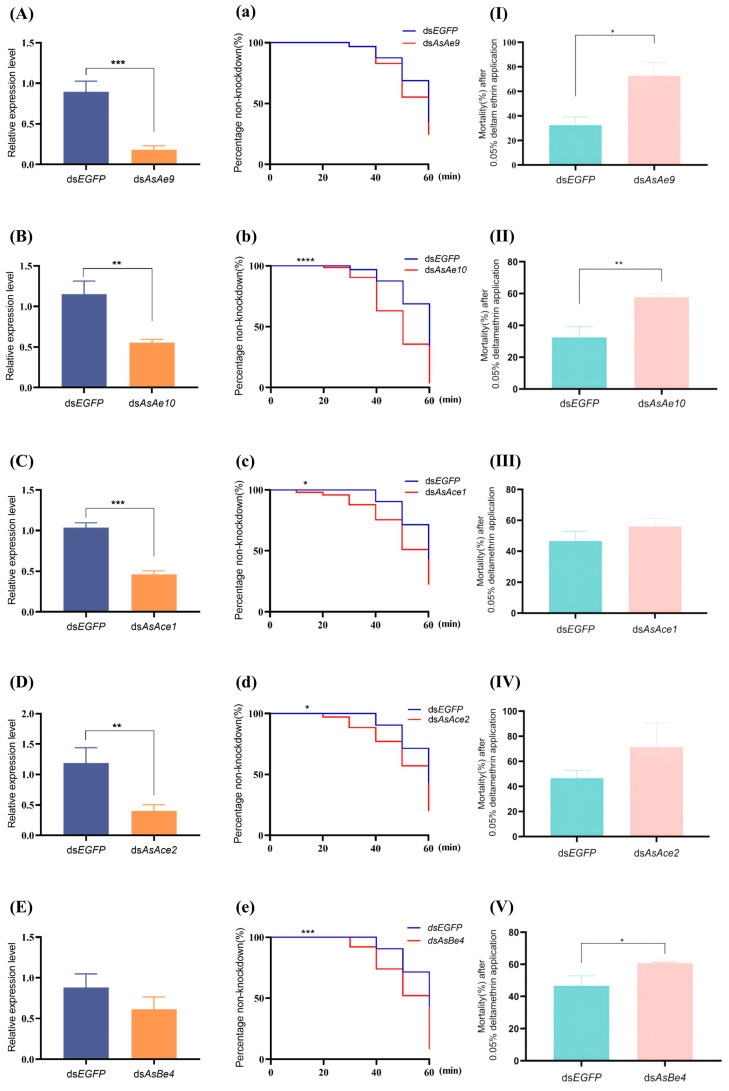
Functional validation of the carboxylesterase genes *AsAe9* (**A**,**a**,**I**), *AsAe10* (**B**,**b**,**II**), *AsAce1* (**C**,**c**,**III**), *AsAce2* (**D**,**d**,**IV**), and *AsBe4* (**E**,**e**,**V**) of *An. sinensis* in its deltamethrin resistance. (**A**–**E**) The expression levels of *AsAe9*, *AsAe10*, *AsAce1*, *AsAce2*, and *AsBe4*post-dsRNA injection (qRT-PCR; mean ± SD). (**a**–**e**) Time–knockdown profiles of *An. sinensis* during 0.05% deltamethrin exposure. (**I**–**V**) Mortality of *An. sinensis* after 24 h recovery following 0.05% deltamethrin exposure (mean ± SD). The groups injected with dsRNA of enhanced green fluorescent protein (*dsEGFP*) were negative controls. The increases in knockdown number were recorded at 10 min intervals for 1 h. The asterisks indicate significant differences between groups (*: *p* < 0.05; **: *p* < 0.01; ***: *p* < 0.001; ****: *p* < 0.0001).

RNAi experiments targeting the genes *AsAe9*, *AsAe10*, *AsAce1*, *AsAce2*, and *AsBe4* in the deltamethrin-resistant strain WX-LR revealed that the relative expression levels of the first four genes were significantly reduced (*p* < 0.01; Figure 4A–D), with silencing efficiencies of 79.8%, 51.8%, 55.4%, and 66.3%, respectively. In contrast, silencing of *AsBe4* did not result in a significant reduction in expression, showing a 30.4% decrease (Figure 4E). In the subsequent bioassay, silencing of *AsAe10*, *AsAce1*, *AsAce2*, and *AsBe4* resulted in significantly shortened knockdown times (*p* < 0.05; Figure 4b–e) in resistant *An. sinensis* following 1 h of exposure to 0.05% deltamethrin. The most significant reduction in knockdown time occurred in the *AsAe10*-silenced group, followed by *AsBe4* (Figure 4b–e). Integrated analysis of the knockdown data revealed that silencing each of the five genes resulted in a reduction in KT_50_ in resistant mosquitoes. Compared to the control groups, gene-silenced *An. sinensis* exhibited reductions in KT_50_ values: *AsAe9* and *AsAe10* decreased from a common control value of 55.12 min (95% fiducial limit [FL]: 53.73–56.66) to 51.09 min (95% FL: 50.36–51.82) and 43.83 min (95% FL: 40.91–46.74), while *AsAce1*, *AsAce2*, and *AsBe4*—sharing a common control value of 57.43 min (95% FL: 56.77–58.15)—declined to 49.09 min (95% FL: 46.27–52.08), 50.08 min (95% FL: 46.16–54.26), and 48.02 min (95% FL: 43.63–52.23), respectively. The non-overlapping 95% fiducial limits between the silenced groups and the control groups indicate a statistically significant reduction in knockdown time. The most significant decrease in KT_50_ was observed in the *AsAe10*-silenced group, which is consistent with the findings described above. RNAi-mediated silencing of five carboxylesterase genes in the pyrethroid-resistant strain enhanced deltamethrin susceptibility, resulting in increased mortality rates of 40.3% (*AsAe9*), 25.3% (*AsAe10*), 9.5% (*AsAce1*), 24.9% (*AsAce2*), and 14.2% (*AsBe4*) after 1 h exposure to deltamethrin followed by 24 h recovery (Figure 4I–V). Silencing of *AsAe9*, *AsAe10*, and *AsBe4* significantly compromised the resistance phenotype, with *AsAe10* exhibiting the most pronounced effect (*p* < 0.05, Figure 4I–V). In conclusion, among the five genes investigated, although silencing of several genes led to reduced knockdown time and increased mortality in the resistant strain after deltamethrin exposure, only silencing of *AsAe10* and *AsBe4* resulted in statistically significant changes in both endpoints. Notably, *AsAe10* silencing produced the most substantial phenotypic alteration. Furthermore, the expression of *AsAe10* was significantly upregulated in the resistant strain, suggesting that it may be strongly associated with pyrethroid resistance in *An. sinensis*. Its overexpression could potentially enhance the metabolic detoxification of deltamethrin in mosquitoes. Interestingly, *AsBe4* silencing also significantly compromised resistance despite its relatively lower silencing efficiency, suggesting its potentially critical role in the resistance phenotype.

Insect esterases can hydrolyze pyrethroids, thereby contributing to detoxification. Enhanced resistance phenotypes correlate with gene mutations that elevate both the expression and hydrolytic activity of these enzymes [68,69]. In *An. gambiae*, transcriptional upregulation of carboxylesterase genes significantly correlates with deltamethrin resistance [38]. In the deltamethrin-resistant *Cx*. *pipiens pallens*, carboxylesterase activity is associated with resistance, with resistance declining concomitantly upon relaxation of deltamethrin selection pressure [39]. In *Ae. aegypti*, resistance to permethrin and deltamethrin has been linked to elevated carboxylesterase activity [40,70], with *CCEae3a* overexpression correlating with deltamethrin resistance [37]. Germline transformation of *Aedes aegypti* with the *An. gambiae CCEae3a* gene—overexpressed in the donor *Ae. Aegypti* strain—elevates enzyme activity and significantly increases α-cypermethrin resistance [51]. In the permethrin-resistant strain of *Cx. quinquefasciatus*, *α-esterase* and *esterase B1* exhibit significant upregulation, respectively, compared to the susceptible stain [15]. Concurrently, three carboxylesterase genes show significant overexpression in the resistant strain, collectively indicating enhanced metabolic detoxification capacity [36]. Carboxylesterase upregulation and enhanced activity represent an important pyrethroid resistance mechanism in mosquitoes. This study confirms that the significantly upregulated carboxylesterase genes in resistant *An. sinensis* are involved in deltamethrin resistance. Similar roles of carboxylesterases in organophosphorus insecticide resistance have been demonstrated. In *Ae. aegypti* and *Aedes albopictus*, temephos resistance is involved in increased carboxylesterase activity, with resistant strains exhibiting significant overexpression of *CCEae6a*, and concomitant gene amplification [71,72].

RNAi-mediated silencing provides a robust approach for functionally validating the contribution of upregulated carboxylesterase genes to insecticide resistance, a methodology extensively applied in resistance mechanism research [73]. RNAi-based functional validation has elucidated the roles of cytochrome P450 in *An. gambiae*, the GST in *Ae. aegypti*, and G-protein-coupled receptor (GPCR) genes in *Cx. quinquefasciatus* permethrin resistance [74,75,76]. In *P. xylostella*, the carboxylesterase genes *PxαE8*, *PxαE14*, and *PxEst-6* exhibit tissue-specific expression in the midgut or Malpighian tubules, and their association with pyrethroid resistance has been validated through RNAi-mediated gene silencing [35,66,77]. Pyrethroid-induced carboxylesterase upregulation (*EoCarE592* in *Ectropis obliqua*; four genes in *Grapholita molesta*) enhances insect susceptibility upon RNAi-mediated gene silencing [78,79]. Additionally, researchers also have considered carboxylesterase-mediated sequestration of organophosphates as an important mechanism for mosquito resistance [80], and this sequestration is closely associated with its overexpression [81]. Currently, novel RNAi-based insecticides have been commercialized for agricultural pest control [82,83]. Multiple studies have confirmed the feasibility of applying RNAi technology for mosquito management [84], with ongoing research focused on identifying mosquito-specific target genes and optimizing dsRNA delivery methods [85,86]. Prospectively, leveraging the insecticide resistance-associated genes identified in *An. sinensis* in this study could enable the development of RNAi biopesticides. These agents would reduce carboxylesterase-mediated metabolism of pyrethroids in mosquitoes, thereby delaying resistance evolution. Furthermore, synergistic application with conventional insecticides could extend their effective lifespan.

This study advances our understanding of pyrethroid resistance mechanisms in *An. sinensis*, and provides a theoretical foundation for optimizing mosquito control strategies. The carboxylesterase genes identified in this research offer molecular tools for resistance management. For *AsAe10*, future studies will bridge mechanism and application. Bidirectional in vivo validation via transgenic *An. sinensis* (overexpression/knockout) will delineate carboxylesterase-mediated resistance pathways, while integrated in vitro modeling (molecular docking/eukaryotic expression) will analyze metabolic dynamics. It is also a significant strategy to develop synergistic *dsRNA*–insecticide combinations for delaying vector resistance.

## 4. Conclusions

This study integrated transcriptomic screening and functional validation to identify carboxylesterase genes involved in deltamethrin resistance in *Anopheles sinensis*. Based on comparative transcriptomic analyses between resistant and susceptible strains, *AsAe9*, *AsAe10*, *AsAce2*, and *AsUn5* were identified as significantly upregulated carboxylesterase genes exhibiting differential spatiotemporal expression patterns and post-blood meal induction profiles. qRT-PCR confirmed significant overexpression of *AsAe10* and *AsAce2* in all four resistant strains, with *AsAe9*, *AsAce1*, and *AsBe4* elevated in two strains. RNA interference targeting the five carboxylesterase genes revealed that silencing of *AsAe10* and *AsBe4* significantly shortened the knockdown time and increased 24 h mortality in the resistant strain upon exposure to 0.05% deltamethrin, with *AsAe10* exhibiting a more pronounced effect. This study identifies these genes as contributors to pyrethroid resistance in *An. sinensis*, provides further insight into the resistance mechanisms, and offers a theoretical basis for refining resistance management strategies against mosquitoes.

## Data Availability

The data presented in the study are available in “FigShare” at https://www.doi.org/10.6084/m9.figshare.29975380.

## Supplementary Materials

The following supporting information can be downloaded at https://www.mdpi.com/article/10.3390/insects16090938/s1. Supplementary File S1: Primer sequences and coding sequences of genes.
