# Supplementary material for: Transcriptome and Functional Analyses Revealed the Carboxylesterase Genes Involved in Pyrethroid Resistance in Anopheles sinensis (Diptera: Culicidae)
Source: Insects. 2025 Sep 5;16(9):938. doi: 10.3390/insects16090938 (PMC12470664; doi:10.3390/insects16090938)
Supplement: Supplementary file 1 [file insects-16-00938-s001.zip › insects-3795878-supplementary.pdf]

1. Primers used for qRT-PCR and RNAi

Primers	Primer sequences (5′-3′)
qrt-*AsAe*4	F: TGGGGGCGATTTGATGT<br>R: TGGGTGACCGTGCTTGG
qrt-*AsAe*9	F: CTATCTTCCACTTCCCGTTCACTG<br>R: CGGTCTGCACCTCGCTCTTTTA
qrt-*AsAe*10	F: GCCATCAGAACGGAACGGAG<br>R: TCGTGCTGCCAGTGTAGAAACC
qrt-*AsBe*4	F: GGTCCCATAGTCGGAGAACAGAG<br>R: TCACTCCACCGCTCGTCATT
qrt-*AsAce*1	F: TTGGGAGAGTGGAAACGACG<br>R: AGAAGTAATAGTGGACCGACGC
qrt-*AsAce*2	F: GAGGCAGTGTGCCTTCTGGA<br>R: GTGTGTAGGTGAATGCGGCG
qrt-*AsUn*4	F: ACATCACGAACTTTGGGGGA<br>R: GTCGGCGTCATAGTCATAACC
qrt-*AsUn*5	F: GACGGTTGGTCTGGAGGGTT<br>R: CGTCCCAGTTGCTCGGAATCT
*RPL*49	F: GGAGCCGGTCGGTGATATGT<br>R: TTCCTTCTCGGTCGGCTTCG
ds*EGFP*-T7	F: TAATACGACTCACTATAGGGAGACGTAAACGGCCACAAG<br>R: TAATACGACTCACTATAGGGAGAGCTCAGGTAGTGGTTGT
ds*AsAe*9-T7	F: TAATACGACTCACTATAGGGAGACTGGTGACCCGAACAACGTA<br>R: TAATACGACTCACTATAGGGAGACGAAACGGTCCGATAGGAGG
ds*AsAe*10-T7	F: TAATACGACTCACTATAGGGAGAGTTCGAGGAGGCGGAAAAGA<br>R: TAATACGACTCACTATAGGGAGAAGCACGGACACTTGATCGTT
ds*AsBe*4-T7	F: TAATACGACTCACTATAGGGAGAAGTTGAATCCCCACCCGTTC<br>R: TAATACGACTCACTATAGGGAGAGGACACCCCAAGAAGTCCAG
ds*AsAce*1-T7	F: TAATACGACTCACTATAGGGAGAACGGTGGCTGAAGGATAACG<br>R: TAATACGACTCACTATAGGGAGACCTGGTGCTGGTTCTGGTAG
ds*AsAce*2-T7	F: TAATACGACTCACTATAGGGAGAGATCAGAACCTCGCCCTACG<br>R: TAATACGACTCACTATAGGGAGATGTAGACGTTGTTGCCCTCC

2. The coding sequence of genes

>AsAe9

ATGGTTATGCGAAATGTTCACAAGCATAGTTGTCTAGGGAACGGAACGCAGCAGTACG
ATGCTTCCTTTCGAGTGATGGTGAAGGTGGTACAGGGGTCAATTTACGGTACGAAGGA
TCGCCTCCCGAACGGACAGCCATACTACTGCTTCAAAGGTATCCCCTACGCTCAACCTC
CGATCGGAAAGCTTCGATTCGCATCTCCGGTGGCTATCGAAAGATACAACGTGTCGTAC
CTGGACTGTAGCCGCGAACGGAGCAACTGTTTGGGTCGAGATGTGATTACTCGCGAAA
TTTCCGGGTCCGAGGATGGTCTGTTCTTAAATGTCTATACACCGCGAATCGTTCGAGAT
GGTTCGCCGGGCGGACTGCTGCCGGTGATGGTGTTCTTCCATGGTGGTGGAATGACGG
GTGGCAATGGAGGAAGCGGCCTCTACCTGCCGGACTATCTGGTGCAGGAGGGTGTGGT
GGTTGCCACGGTTAACTATCGTCTGGGAGTGCTTGGATTCCTGTGTCTACCACGTGCGG
GCATTGAAGGGAACGCTGGGCTGAAGGATCAGCGTATGGCGTTGCAGTGGATCGCTCA
GAATATAAAAACGTTCGCTGGTGACCCGAACAACGTAACCATCTTTGGGGCCAGCTCG
GGTGGAAGCAACGTCATGATGCACTGCTTTTCCGAACTTTCGAGACCATACTTTCACA
AGGCGATTGCGCAGAGTGCGACCGTGTTTGCGGATCTGATTTACCAAAGCGAGCCGGA
GAATCGGGCTCGTTCTCTGGCGCGCATCTTCGGATACGAGGGTTCATCGGACGAGGGA
GCACTGGCAACACTGCGGGACGTACCGGCACACCGTCTCTACGAGGCACAGTTTCTTG
TACTGTCCGATCGAGAGCGTGAGTATGGACCTATCTTCCACTTCCCGTTCACTGCCGTC
ATTGAGCGGAAGGAGTCCCGGGATGCCGTTTTGATCAAGACTCCCATGGACTATCTAA
AAGAGCGAGGTAGCATACCGGTACCGGTTCTCTGCGGATATAACGATAAGGAGGGCAT
CATGGAGCTGGTAGACACGATCAAAACAATCTCCGTGTACAACCGCCAGCCGGAGAA
GTTCATCTGTGGCTCGTTCAATGTGGACTACTTTAGCCCGCTTGCTCGAGCCCTCGGGG
AGGAGTTGAAGCGACACTACTTTGGCGATCAGCTGATCACACGTGACAACCTGCACCA
CATGGTGGACCTCCTATCGGACCGTTTCGTTGTAGGATACTACGTGCTTTGCCAGCTGT
GGAGTACCTATCAGCCTCAGACACCGTTCTACTCGTACCGTTTCGCTTTCGATGGCTCG
TTGAACAAGGGCAAGGAGTTGCTTAAGTTCCAGCAACTTCCGGGCGCTTGCCACATCG
ACGAGGTGTACTACATCTTCAGCTCACCGTTGCTTCGGACGGAAATTCCACCGACCGA
CCAGGCGTATGCGATGCGCCAAACGATGGTGCGCATGTGGACCAACTTCGCCAAGTAC
TCCGATCCTACGCCAGCGAACGATTTAGCTCGGCTTCCGTGCCGCTGGGAACCGATGG
TCGCGGTGCCACAGGGTGAAGAAACCCCACCCAACTACACCCTGCTTGCGATCGGTAA
CGAAGAGGTGAAGATGGACACACTGCCGGAGTTGCAACGGTTGGCCAAATTTCTAGA
CATCCTTAAGCGATGCAATGGGTCAATAGATAACTTCGTTATACCGCGTGTTGATGCTTG
A

>AsAe10

ATGGTTATCCGCAAGCTGTACGTTCCGCCGACGGTCGTAAAGAAAGATCCAGTTCCGC
CGCGCGTGGATCCGAACTCTTTGCCTCCGGCCACCGCCTATCAGCCCCCTTCCAGCGAT
CTACCGTTGATCGTCGACGAGGCGAGTGTGCCGAATGGCGGTGAACCGTTGGTTTTTG
CGGACGATGTCGAGCTTACCATCAAGCCGGGGAAGATTGTAGGCCGCCGGAAGGTGA
GTGTGGCTAACAGGGAGTACTACTCCTACCAGGGTATTCCGTACGCGGAACCGCCCGT
GGGTGAGCTGCGCTTTAAGCCACCGGTTCCGGTGGAAAAGTTTGCGGAAGAACCGCT
GGACTGTAGCCAAGAGCGGGGTATCTGCACTGCCTGTTTGTACCTGCCGGAAAGCCAT

CAGAACGGAACGGAGGATTGTCTCTATCTGAACGTGTACACAACGAGTGCTCCTAGCG
GTCAGCCCGGTGTGCTGATGCCGGTCATGGTGTGGATCCATGGTGGCGGTTTCTACACT
GGCAGCTCGAGCACGACTTTCTACGGTCCGGAGTATCTGGTGCAGGAGGGCGTGGTCT
TGGTGACGTTCAACTATCGCCTGGGACCGCTCGGATTCCTCGCCCTTCCGTCCGCGGGC
ATCCACGGGAACCAGGGCCTGAAGGACCAACAGCTGGCTCTTCGCTGGGTGAAGGAG
AATATCGTACATTTCGGTGGCGATCCATCGAACGTGACGCTGTTCGGGGAAAGTGCTG
GCAGTGCTTCGGTTAACTGGCACTACCTCTCGCCAACGTCTCGCCAGTTCTTCCACAA
GGCAATCTGCCAGTCGGGCTCGGTGTTCTGCCCCTGGGGGCTGCAGTACCAACCGGAG
CATAAGGCACGCAAGTTGGCCGCCGTGTTGGGCTACGAGGGAAACAATGATGCGGGA
GTTTTGGAAACGCTGCGAAACGCTTCGGTCGAAGATTTGGTCGCGAACGCTAGCAAA
GCGTTCGAGGAGGCGGAAAAGAGCATCTACTTTGTGTCACCGTTCATTCCCTCGATCG
AGGACGTCACGTCGGAGGATCCGATCATTCCGCAGCGTGGTGAGGAGCTTCTGAAGC
AACCCAACGTGACATCGATTCCCCTGATACATGGGGTGACCAGTGGCGAGGGGTTGGT
CATGTATGGACGCCTGCTGACGATGCTGGACGAGGTGGCGGGTAACTTGAGCATGGTT
CTGCCGCTAGACTTCGGAGTCCCTCGCGATACGGCACCAGCCATATTGGACGAGATTCG
TCAGTTCTACTTCCAGGATCAACCGATCGGCCACGATACGCTTCTACGCGTGGTGGATC
TTTTGGCAGACACCGGTTTCAATTTCCCCGTGTACAATGCCGCTGAGCTGCATTCTCGC
TATCAGCAGGAAGCCCCACTGTACTTCTACTATTTCGCGTACGAAAACGAGCTAAACCA
GATGCGCAAGCTCTTCAACGTACCGGAAGGAACCCCGGGAGCGGCCCACGCTGACGA
ACTTCCGTACCTGTTCAGTGGTTCCGAGTACGGCGTGCAGGTCGAGCCGGATTCCAGC
GCCGGCCGAGCTCGATCGCTACTGTGCAAACTTTGGACTAACTTTGCCAAGTTCGGCA
ACCCAACGCCGGACGAGCAGGTCGACAGCATCGGCTTCCGGTGGGAACCGATCGCCC
CCACACCCTCCGGGGAACCGTTCGTCCTGAGGGCCCTCGAGCTGAACGATCAAGTGTC
CGTGCTGGAGAATCCGTTGCACGATCGGATACAGTTTTGGAAGTCGCTGGCCGAACGC
TACAACCCGACACTGCTAGGCTAA

＞AsAce1
ATGACTAGCAACCACTCCGTCTGCCGGCGATCGTGCGCCAACGTGCTGGTGCTGCTGC
TCGGTGCGACCGTGATATGTCCGGCGTACGGCATCATCGACCGGCTGGTGGTGCAGAC
CAGCAGTGGACCAATACGCGGCCGCTCGACCATGGTCCAGGGGCGCGAGGTGCATGT
CTTCAATGGGGTGCCGTTCGCCAAACCGCCGGTCGACGGGCTGCGGTTCAAGAAGCC
GGTCCCCGCCGAGCCGTGGCACGGAGTTCTCGATGCAACCAGATTACCTCCATCGTGC
ATTCAGGAACGGTATGAGTATTTCCCCGGCTTCGCTGGCGAAGAGATGTGGAATCCCA
ATACCAACGTTTCTGAAGATTGTCTGTATCTGAACATCTGGGTGCCAACCAAAACTCGT
CTCCGACATGGCAGGGGTCTGAACTTTGGCAGCAACGACTACTTCCAGGATGACGAGG
ACTTTCAGCGCCAGCACCAATCGAAGGGTGGTCTGGCGATGCTAGTGTGGATCTACGG
GGGTGGCTTCATGAGTGGCACCTCCACGCTGGACATCTACAACGCCGAGATACTGGCG
GCCGTCGGTAACGTCATCGTAGCCTCGATGCAGTATCGCGTGGGCGCGTTCGGTTTCCT

CTACCTGGCACCGTACATCAACGGCTACGAGGAGGACGCTCCGGGAAACATGGGTTTG
TGGGATCAGGCGCTAGCGATACGGTGGCTGAAGGATAACGCCAAGGCGTTCGGTGGCG
ACCCGGATCTCATCACGCTGTTCGGTGAGTCGGCTGGCGGTAGCTCGGTCAGTCTGCA
CCTGATGTCGCCCGTCACCAGAGGACTGTCCCGGCGTGGCATTCTGCAGTCGGGCACG
CTGAACGCCCCGTGGAGCCACATGACCGCCGAAAAAGCGCTGCACATTGCCGAGGGC
CTAATCGACGACTGCAACTGTAACCTCACGATGCTGAAGGACTCGCCCAGCACGGTCA
TGCAGTGCATGCGGAATGTCGACGCCAAAACAATCTCCGTGCAGCAGTGGAACTCCTA
CTCCGGCATACTGGGATTCCCGTCCGCGCCGACCATCGATGGAGTCTTCATGACCGCGG
ATCCGATGACCATGCTGCGTGAGGCGAACTTGGAAGGAATTGACATCCTGGTCGGTAG
CAATCGCGATGAAGGCACATACTTCCTCCTGTACGACTTTATCGATTACTTTGAGAAGG
ATGCGGCCACCTCGCTACCCAGGGACAAGTTTTTGGAGATCATGAACACCATCTACAA
CAAAGCCTCCGAACCGGAACGCGAAGCCATCATATTCCAGTATACGGGTTGGGAGAGT
GGAAACGACGGCTACCAGAACCAGCACCAGGTGGGCCGGGCGGTCGGCGACCACTTC
TTCATCTGCCCGACCAACGAGTTCGCCCTCGGTCTGACGGAACGCGGCGCGTCGGTCC
ACTATTACTTCTTCACGCACCGCACCAGTACCTCCCTCTGGGGCGAATGGATGGGCGTT
TTGCACGGTGATGAGGTGGAGTACATCTTCGGCCAGCCGATGAACGCGTCGCTGCAGT
ATCGCCAGCGGGAGCGGGACCTGAGCCGGCGTATGGTCCTGTCAGTGAGCGAGTTCG
CACGGACGGGAAACCCGGCACTCGAGGGGGAACACTGGCCGCTCTACACGCGCGAA
AATCCGATCTACTTCATCTTCAACGCCGAAGGGGAGGACGATCTGCGCGGCGAGAAGT
ATGGCCGCGGTCCGATGGCCACCTCGTGCGCCTTCTGGAACGACTTCCTGCCGAGGTT
GCGCGCCTGGTCCGTTCCCTCGAAGACGCCGTGCAACTTGGTCGAGGCCATCTCGCAG
GGCAGCAGAACCGGGGCGACGTTGCTGTACCTCGTAGCGATGCTGGTCTTAGTACTGT
CCCTGCGCCAACCGGCGCTTGTGAACTAA

＞AsAce2
ATGGAGATCCGAGGGCTCCTGATGGGCAGGCTGCGGTTAGGTGGCCGCCAGGTGGCG
CCTCTGCTGAGTCTGTGCACCCTCGCCCTGCTCGCCCCGTCCCTGCTGGTGCCGGTGG
TCCACGGACGGCACCACGAGCTCAATAATGGCGCCGGTTCGCTCGGGTCGCATCAGCT
GTCGGCGGCCGGTGGCGTTGGCGGCGTTGGCCTATCCTCGTCCCAGCCAGCCGACTCG
ATCTCGGGTGCCGCCGTCGCGGGGGCCGAGGAGGACGTGGGCCGGATTGCTGCCCTC
AGCAAAGACGCAGATGCATTTTTTACACCATATATAGGTCACGGTGAGTCCGTACGAAT
TATAGATCCCGAGCTGGGCACGCTCGAGCGCGAGCATGTCCACGGTGGAGCGACACCG
CGTCGGCGCGGTCTGACGAGACGTGAGTCAAACTCAGATGCCAACGATAATGATCCGC
TGGTGGTGAACACCGACAAGGGTCGCATCCGCGGGTTTACGGCCGAGGCACCGAGCG
GCAAGAAGGTGGACGTGTGGCTCGGCATCCCGTACGCGCAACCGCCGGTCGGACCGT
TGCGCTTCCGACACCCGCGCCCGGCCGAGAAGTGGACCGGTGTGCTGAACACGACGA
CCCCGCCGAACAGCTGCGTGCAGATCGTGGACACCGTGTTCGGTGACTTTCCCGGTGC
GACCATGTGGAACCCCAACACGCCCCTCTCCGAGGACTGCCTGTACATCAACGTGGTG

GCACCCCGGCCCCGTCCGAAGAATGCGGCCGTCATGCTGTGGATCTTCGGCGGTGGCT
TCTACTCCGGCACGGCCACGCTTGACGTCTACGACCACCGGGCGCTGGCGTCCGAGGA
GAACGTGATCGTCGTCTCGCTGCAGTACCGTGTCGCTAGCCTGGGTTTCCTGTTCCTCG
GCACACCGGAAGCACCCGGCAATGCGGGACTTTTCGATCAGAACCTCGCCCTACGATG
GGTTCGAGACAACATTCACAAGTTCGGTGGAGATCCCGCACGAGTGACACTGTTCGGC
GAAAGTGCTGGTGCTGTGTCCGTCTCGCTGCATCTACTGTCCGCACTCTCGCGGGACC
TTTTCCAGCGGGCCATCTTGCAGAGTGGTTCCCCGACGGCACCCTGGGCACTTGTGTC
TCGCGAGGAAGCAACTCTCCGAGCCATTCGGTTGGCGGAAGCGGTTGGATGCCCGCA
CGACACGAGCAAGCTCAGCGAAGCGGTCGAGTGTCTGCGCGGCAAGGACCCGCACGT
GCTGGTCAACAACGAGTGGGGTACGCTCGGCATCTGCGAGTTCCCGTTCGTGCCGGTG
GTGGACGGTGCGTTCCTGGACGAGACGCCGCAGCGGTCGCTCGCGAGCGGGCGCTTC
AAGAAGACGGACATCCTCACCGGCAGCAACACGGAGGAGGGCTACTACTTCATCATCT
ACTACCTGACCGAGCTGCTGCGCAAGGAGGAGGGCGTGACGGTGTCGCGCGAGGAGT
TCCTCCAGGCGGTCCGCGAGCTCAACCCGTACGTCAACGGGGCCGCCCGGCAGGCGA
TCGTGTTCGAGTACACCGACTGGACCGAGCCGGACAACCCGAACAGCAACCGGGACG
CGCTGGACAAGATGGTCGGCGACTACCACTTCACGTGCAACGTGAACGAGTTCGCCC
AGCGGTACGCCGAGGAGGGCAACAACGTCTACATGTACCTGTACACGCACCGGAGCA
AGGGCAACCCGTGGCCGCGCTGGACCGGCGTCATGCACGGCGACGAGATCAACTACG
TGTTCGGGGAACCGCTCAATCCCACCCTCGGCTACACCGACGACGAGAAGGACTTCA
GCCGGAAGATCATGCGATACTGGTCCAACTTTGCCAAGACTGGGTACGTACCGAAGGA
GAAGGAGATCTGTGGGGAGTCGAGGTCTGACGATGTTTTCCAAACCCCAAACAGCAA
TCCAAATCCCAGCCAACTCAGCACCGACTATCCCGAATGGCCCAAGCATACGGCCCAC
GGACGGCACTATCTGGAGCTCGGTCTGAACACGTCCTTCGTCGGGCGGGGCCCACGGT
TGAGGCAGTGTGCCTTCTGGAAGAAGTATCTACCCCAGCTAGTTGCAGCTACCTCTAAC
CTACAAGTGGCACCACCACCAAGTGCACCGTGCGAAAGTAGCGCCTTTTTCTACCGGC
CTGATCTGATCGTACTGATAGTGTCGCTGCTTTCCGCCGCCGCATTCACCTACACACAAT
AA

>AsBe4
ATGAAGGAACCAACGACGGCGTTAGTGCAAGGTTTTGTTGTAATGGTCAGCTTATTGC
TGCTCGTGTTCTTCACAACTGCCAGCGCAACGTTAGTGAACCTCGAGAATGGTCCCAT
AGTCGGAGAACAGAGGGGAGAATACTTCGCGTTCGAGGGCATTCCGTATGCGAAGCC
ACCCGTTGGGAAGCTCCGGTTCGCACCGTCCGAGCTGAATGACGAGCGGTGGAGTGA
ACCGAAAAATGTTACCCGCTTCGGACCGGTTTGCCTGCAGTGGGATCATTTCGTGCCG
GACACGGACAAGCTGGTCGGTGAAGAAGATTGCCTGTTTCTGAATGTGTACACACGG
ACAGTCGACCGGGAGGCCAAACAACCTACGATCGTGTTCATCCACGGCGGAGCGCTG
ATGTTCGGCACCGGAAGTTTCTATCAACCGGATCACGTGATGCGCCGCCCTCTGGTGC
TGGTCACGTTCAACTATCGGCTCGGTCCGCTCGGGTTCCTGAGTACCGAGGACGACGT
GATTCCCGGGAACTTTGGCCTGAAAGATCAAGTGACGGCCCTGCAGTGGGTTAAGAA

AAACATCCACCACTTCGGTGGCGACCCCGAGCGGGTTACAATCGTAGGTTATTCGGC
CGGCTCGGCCAGTGTGCATCTGCACTACCTTTCGCCGCTATCGAGTGGGTTGTTTCG
TCCGGCATCGGTCACAGTGGTTCCGCCCTCAATCCCTGGGTGATGGCGGAACGGTCGC
TGGAAAAAGCGATCCGCATCAGTTCCATTCTTGGCTGTCCGACACGCAAAACCGACC
TCATGCTCGAGTGTTTGCGCAAGCGACCGGCGGAAGATATTGTCCGCCACGTGCCCG
GATTCCTCGACTTCCTGTACAATCCTTTCTCGCCCTTCGGGGTGGTGGTGGAGCACGG
TGGTAAGTTGAATCCCCACCCGTTCCTGGAGGAAACGCCACGCAAGCTTATGACCTC
GGGAAAGTTCACCCACGAGCCGCTGATACTGTCGGTCACGGAGGCCGAAGGGTTGTA
TCCGGGGGCGGAGTTTATCAGCGACCTGAGTCATCTCGAAGCGATCGATCGGAGGTG
GAACGAGCTTTTACCGAGCATTCTGGATTACAAAACGGCCGTCCCCGATTCGAGGCA
GCGGGATCGGCTCTCGGAAGCCATTCGGGCGCATTATTTCGCACCGGGAACGAACCT
ATCCCTGGACAACTTTTCCGATCTCATACGGATTATCTCGAATCGGTTGTACTTCACG
GGAGTGACCGAGTCGGCCAAACTGATGCAACCACACACGGAGGTCTACCTTTACCTG
GACCACTACAAAGCACGGTACGGCGTCGGGGAAGCTTTGTCACATCGGGATGAACCT
GGACTTCTTGGGGTGTCCCATGGCGACGATGTCCTGCTCATCTTCCCCAGCGTCCTTC
GGGAGCTGGTACCCTTCACGGCGGAGGAAATGGAAGTGGTCGATCGGTTTGTGGCCA
TGTACGAAGCGTTTGCCAGCGGACAGAAACCCACGTTCGGTTCGGACGCATTGCCGG
TGCAGGACTCACAGGAAACGGTTACCTTCCTTAAGCTTAACTACCCGACCAGCGAAA
CGGTTCGAGCGAAAGGAGTGAGTGATGAAGAATTTTGGACCAGCCTCGATTTTAACG
ATGCACCGTACGAAATTTTAGCATCTCACGATGAGCTTTAG
